# Mechanisms underlying targeted mitochondrial therapy for programmed cardiac cell death

**DOI:** 10.3389/fphys.2025.1548194

**Published:** 2025-04-11

**Authors:** Fengting Jing, Min Zhao, Hemin Xiong, Xin Zeng, Jun Jiang, Tao Li

**Affiliations:** ^1^ Key Laboratory of Medical Electrophysiology of Ministry of Education, Institute of Cardiovascular Research, Southwest Medical University, Luzhou, Sichuan, China; ^2^ School of Continuing Education, Southwest Medical University, Luzhou, Sichuan, China; ^3^ Department of General Surgery (Thyroid Surgery), Southwest Medical University, Luzhou, Sichuan, China

**Keywords:** mitochondria, cardiomyocyte, PCD, ferroptosis, autophagy, apoptosis, pyroptosis, cuproptosis

## Abstract

Heart diseases are common clinical diseases, such as cardiac fibrosis, heart failure, hypertension and arrhythmia. Globally, the incidence rate and mortality of heart diseases are increasing by years. The main mechanism of heart disease is related to the cellular state. Mitochondrion is the organ of cellular energy supply, participating in various signal transduction pathways and playing a vital role in the occurrence and development of heart disease. This review summarizes the cell death patterns and molecular mechanisms associated with heart disease and mitochondrial dysfunction.

## 1 Introduction

The heart is an organ that has an essential role in human body, with its primary functions to pump blood, provide pressure for blood flow, and supply oxygen and nutrients to cells throughout the body to maintain life activities. Mitochondria are abundant in myocardiocytes, and mitochondrial quality control affects cardiac function by regulating the state of myocardiocytes.

Mitochondria are organelles that comprise a self-proliferating bilayer membranal structure in eukaryotic cells. The structure of mitochondrion consists of an outer mitochondrial membrane (OMM), mitochondrial intermembrane space (IMS), mitochondrial inner membrane (IMM) and mitochondrial matrix (Lin et al., 2021). The OMM is a smooth and elastic membrane without folds, and is distributed with many pore proteins and modified proteins that limit the penetration of the membrane by macromolecular (Chu Q. et al., 2023). Proteins on the OMM participate in maintaining mitochondrial quality control and cellular lipid and carbohydrate homeostasis (Tong et al., 2018). The IMS, which located between the OMM and IMM, contains enzymes and ions that maintain mitochondrial function and osmotic pressure. The IMM is composed of a large number of cardiac phospholipids (Luévano-Martínez et al., 2022), with a lack of pore proteins in the IMM ensuring low permeability. The transmembrane movement of small-molecule compounds and ions such as H^+^, ATP, and pyruvate are thereby strictly regulated, as they cannot freely penetrate the IMM and must be assisted by a carrier. Hence, there exist several permeability enzyme shuttles in circulatory system (Gutiérrez-Fernández et al., 2020). The IMM also sustains a specific mitochondrial membrane potential (MMP). The IMM reflects a deep invagination as its topological structure, and the respiratory electron transport chain (ETC) is used for oxidative phosphorylation (OXPHOS) (Gutiérrez-Fernández et al., 2020; Singh et al., 2023). The IMM morphology is produced and supported by mitochondrial contact sites, ATP synthase, fusion protein, phospholipids and phosphatidylethanolamine (Stephan et al., 2020; Luévano-Martínez et al., 2022). The mitochondrial matrix is the site of various metabolic reactions, including the tricarboxylic acid cycle (TCA) (Zhang Y. et al., 2020), fatty acid beta oxidation (Cui X. et al., 2023), Ca^2+^ signal transduction (Quarato et al., 2022), and the biosynthesis of heme, phospholipids, and other metabolites. These metabolites then regulate cellular life activities such as cell growth and development, biosynthesis, physiological activities, and cellular aging and apoptosis.

**FIGURE 1:**
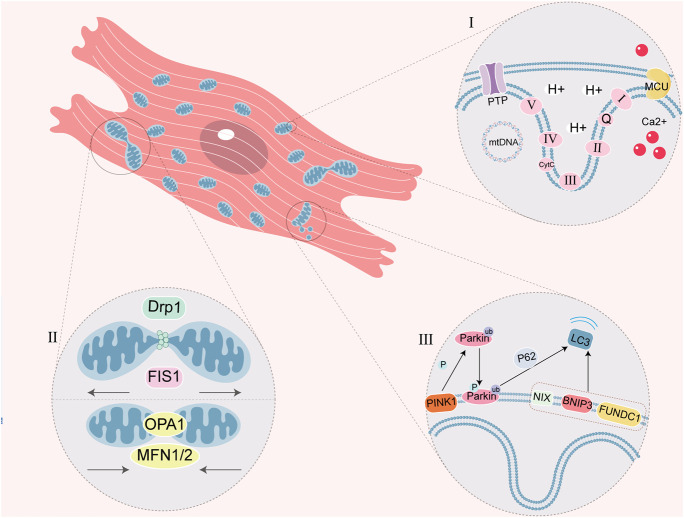
Schematic diagram of myocardiocyte mitochondrial function. **(I)** ETC is a series of complexes used for mitochondrial respiratory function. PTP closes in physiological conditions and prohibits macromolecule shuttle, but it mediates the formation of MPTP in mitochondrial dysfunction. MCU is one of Ca^2+^ single transporter to regulate the Ca^2+^ concentration in the mitochondria, which Ca^2+^ overload cause MPTP. Circular mtDNA mainly encodes the protein of mitochondrial OXPHOS. **(Ⅱ)** Mitochondrial fission and fusion is a link of mitochondrial quality control. DRP1 and FIS1 regulate fission, OPA1 and MFN1/2 regulate fusion. **(Ⅲ)** Mitophagy is a physiological process, which mediated by ubiquitin ligase Parkin and PINK1 or mitophagy receptor (NIX, BNIP3 and FUNDC1). P62 is the ubiquitin-binding protein that promotes Parkin-dependent mitophagy.

There is independent genetic material called mitochondrial DNA (mtDNA) in the mitochondrial matrix. The synthesis of proteins is regulated by both nuclear DNA (nDNA) and mtDNA in mitochondria. Most mitochondrial proteins are encoded by nDNA and undergo processes such as transcription, translation, and modification in cytoplasm, translocating into mitochondria to perform specific function (Fan et al., 2019). Other parts of mitochondrial proteins are independently encoded and synthesized by mtDNA, and play an important role in mitochondrial quality control. Previously, mtDNA was believed to be circular and capable of encoding 13 peptides, 22 transfer RNAs (tRNAs) and 2 ribosomal RNAs (rRNAs), which participate in the OXPHOS process through ETC (Xu et al., 2019). Recently discovered 14th protein CYTB-187AA encoded by mtDNA, which is located in the mitochondrial matrix, has been found to interact with membrane carrier and regulate ATP synthesis. But its physiological function remains to be explored (Hu et al., 2024; Figure 1).

Mitochondria not only synthesize ATP through OXPHOS to provide energy for cells, but also participate in multiple signaling pathways and regulate cellular activities. The maintenance of mitochondrial functions relies on quality control of mitochondria, including dynamic balance of mitochondrial generation, division, fusion and autophagy (D'Acunzo et al., 2019; Pfanner et al., 2019; Tan et al., 2022). When the function of mitochondria decreases, mitochondria divide into dysfunctional fragments which engulfed by lysosomes. Remaining fragments will undergo fusion to shared mtDNA, ETC and other internal structures, which maintain the function of mitochondria (D'Acunzo et al., 2019; Jeong et al., 2022; Tan et al., 2022).

## 2 Mitochondrial disorders

Mitochondria are organelles that are highly sensitive to cellular homeostasis, and there are numerous injurious factors that lead to severe functional impairments. These impairments can be manifested as decreased mitochondrial synthesis, reduced mitochondrial metabolic capacity, and imbalanced mitochondrial quality control. The basic mechanisms of mitochondrial damage include mtDNA damage, mitochondrial membrane damage, and various metabolic disorders and imbalances in the production and clearance of reactive oxygen species (ROS) (Shen et al., 2019).

### 2.1 Mitochondrial DNA damage

Mitochondrial proteins that are encoded by mtDNA in cells, are related to oxidative phosphorylation, and this process can be easily influenced by the external environment and genetic factors, leading to mutations that generate mitochondrially related diseases (Lehmann et al., 2019). mtDNA is also susceptible to the influence of exogenous substances, such as exposure to physicochemical or biological factors, and some endogenous factors, such as DNA replication errors, DNA instability, and ETC production of ROS (Ziada et al., 2019). Continuous biochemical reactions in the mitochondria readily produce harmful substances that include ROS, and mtDNA lacks histone protection, and thus mtDNA can be damaged by ROS attacks. The accumulation of mtDNA damage can also affect the synthesis of OXPHOS-related proteins that will lead to mitochondrial dysfunction (Filograna et al., 2019). Therefore, promoting the repair of mtDNA is beneficial to restoring mitochondrial function. Mitochondrial fusion can also eliminate the heterogeneity of mtDNA. mtDNA damage reduces ATP synthesis and activates mitochondrial autophagy. Mitochondrial autophagy is activated depending upon proteins such as PINK1, Parkin, ULK1 (Chinnery and Prudent, 2019; Jiménez-Loygorri et al., 2024), BNIP3/NIX and FUNDC1 (Baechler et al., 2019) that counter damage repair. For example, sirtuins are a deacetylases family evolutionarily conserved NAD-dependent histone, closely related to biological functions such as mitochondrial energy homeostasis, antioxidant activity, proliferation, and DNA repair (Zheng et al., 2021). The activity and expression of Sirtuin 3 in mitochondria can affect oxidative metabolism efficiency, regulate mitochondria biogenesis, enhance mitochondria function, and coordinate repair of mtDNA by oxidative damage (Park et al., 2020). Targeting Sirtuins can improve mitochondrial function in myocardiocyte injury (She et al., 2023; Yang Z. et al., 2024).

### 2.2 Decreased mitochondrial respiratory capacity/decreased membrane potential

When cells are under hypoxia, OXPHOS is inhibited, ATP synthesis is reduced, mitochondrial oxygen consumption is attenuated, and intracellular biosynthesis is affected. However, studies have shown that a moderate reduction in MMP could be employed to induce antibacterial stress in yeast strain (Liu et al., 2021). Under physiologic circumstances, the hydrogen carrier NADH and FADH2 transfer electrons, which combine with oxygen to form water at complex IV. Pathologically, superoxide anions are generated, producing a large number of harmful substances, including ROS. Specific transmembrane proteins located on the mitochondrial membrane restore mitochondrial respiratory capacity and maintain membrane potential. Targeting mitochondria this protein improved mitochondrial function in heart failure patients (Tang et al., 2023).

### 2.3 Imbalance in mitochondrial quality control

Changes in the cellular environment can disrupt the dynamic balance between mitochondrial fusion and fission under pathological conditions, triggering changes in protein function or activity that affect mitochondrial quality control (Nie et al., 2021). Mitochondrial division is principally mediated by dynamic related protein 1 (DRP1), mitochondrial fission protein 1 (FIS1), and mitochondrial fission factor (MFF) (Chang et al., 2022). The fusion process is divided into OMM fusion and IMM fusion, and they are respectively mediated by mitochondrial fusion protein (MFN) and optic atrophy type 1 (OPA1) (Hu et al., 2020). The morphology, number and size of mitochondria are closely related to the metabolic status of cells. When mitochondrial function is abnormal, the cellular energy supply is insufficient, proteins related to division are upregulated, and proteins related to fusion are downregulated (Smith et al., 2019). Mitochondrial aminoacyl-tRNA synthetase regulates the translation of proteins within mitochondria. Synthetase deficiency inhibits mitochondrial fusion and promotes mitochondrial division, which alters mitochondrial dynamics and causes hypertrophic cardiomyopathy (Li B. et al., 2024).

### 2.4 Mitochondrial calcium overload

Calcium homeostasis is related to cardiomyocyte contractions, rhythm and electrical activity, and mitochondria are important in the calcium-concentration buffering system. Pathological conditions, such as oxidative stress caused by ischemia-reperfusion, abnormal sodium calcium exchange, and protein kinase activation can then create cellular calcium overload. The regulation of mitochondrial calcium homeostasis is related to the mitochondrial calcium monotransporter protein (MCU) localized at the IMM, which allows Ca^2+^ to enter the mitochondrial matrix (Quarato et al., 2022). Obesity-related studies have shown that the mitochondrial autophagy receptor FUNDC1 interacted with the related ubiquitin ligase complex receptor, the subunit of which regulated IP3R2 receptor degradation, preventing calcium overload and alleviating myocardial lipotoxic damage (Ren et al., 2020).

In summary, mitochondrial dysfunction is widely reported in various heart diseases, and different heart diseases can be due to the same mitochondrial oxidative stress-damage mechanism the same heart disease can also be regulated by different mitochondrial targets. Thus, macroscopically, heart disease is closely related to mitochondrial function.

## 3 Heart disease and programmed cell death (PCD)

Programmed cell death (PCD) refers to the process of molecular programmed cell suicide mediated by specific genes within cells, and the process is related to the growth and development of multicellular organisms, as well as to the homeostasis within the organism. PCD involves multiple aspects and conditions, including immunity, cancer and infection (Nguyen et al., 2023). PCD comprises ferroptosis, apoptosis, autophagy, pyroptosis, and the recently uncovered cuproptosis.

### 3.1 Mitochondrial dysfunction and ferroptosis

**FIGURE 2:**
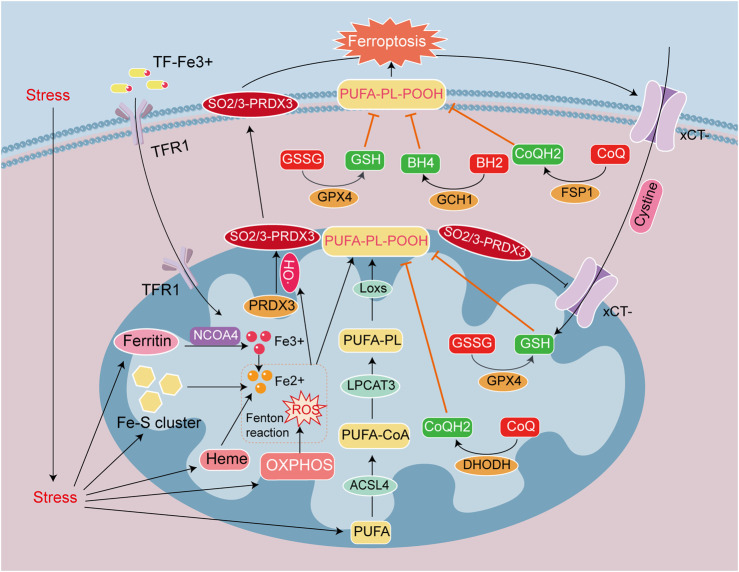
Signaling mechanisms leading to ferroptosis in heart disease. Ferroptosis is caused by the accumulation of iron ions and lipid peroxides, and the destruction of the balance of the antioxidant system. Iron deposition is mainly caused by TF-Fe^3+^ entry into cells through TFR1, and the autophagic breakdown of Ferritin, Heme, and Fe-S cluster (NCOA4 mediates ferritin autophagy). Lipid peroxide deposition is due to the Fenton reaction between Fe^2+^ and ROS to form HO^**•**^. PUFA converted to PL-PUFA-OOH via ACSL4, LPCAT3 and LOX with HO^**•**^, and PL-PUFA-OOH accumulate on the cell membrane make ferroptosis. The antioxidant system GSH/GSSG/GPX4, CoQH2/CoQ/DHODH, CoQH2/CoQ/FSP1 and BH4/BH2/GCH1 prevent the generation of PUFA-PL-POOH. PRDX3 is an antioxidant enzyme in mitochondria, but it is attacked and formed SO2/3-PRDX3 by HO^**•**^. SO2/3-PRDX3 is a specific marker of ferroptosis, and it suppresses xCT-cysteine uptake, reduces the antioxidant capacity of GPX4 and GSH/GSSG, and promotes ferroptosis.

Ferroptosis is a novel form of PCD characterized by iron overload and ROS-dependent accumulation of lipid peroxides (Dixon et al., 2012; Stockwell, 2019), and is related to iron metabolism and lipid metabolism disorders. The generation and accumulation of membrane lipid peroxides eventually precipitate cell membrane damage and rupture (Riegman et al., 2020). Cellular ferroptosis can be regulated through iron metabolism, lipid metabolism, and antioxidant systems (Chen et al., 2020; Deng et al., 2020). Mitochondria are the chief organelles involved in the utilization, catabolism, and synthesis of iron and lipids. A recent study confirmed that the ferroptosis-specific indicator, peroxide-PRDX3 (SO2/3-PRDX3), is a mitochondrion-specific peroxidase that is a member of the PRDX antioxidant enzyme family. During the induction of ferroptosis, PRDX3 either underwent excessive oxidation due to exposure to mitochondrial lipid peroxides, and SO2/3-PRDX3 subsequently translocated from the mitochondria to the plasma membrane, or mitochondrial PRDX3 might reposition itself to the plasma membrane and then underwent excessive oxidation on the membrane. The presence of SO2/3-PRDX3 on the plasma membrane ultimately inhibited the uptake of cysteine, thereby promoting ferroptosis (Cui S. et al., 2023; Figure 2).

Disorders of iron metabolism are manifested by an increase in ferrous ions in the unstable iron pool. It is related to iron transport, ferritin autophagy and iron sulfur clusters protein degradation in mitochondrion (Chen et al., 2020). Ferroptotic inhibitors Fer-1 and deferoxamine DFO may then be used to reduce intracellular levels of ferrous ions, thereby inhibiting ferroptosis (Guo et al., 2022). Disorders of lipid metabolism are reflected in the generation of lipid peroxides, and we acknowledge that polyunsaturated fatty acids (PUFAs) are components of cell membranes and membranous organelles. The C-H bond located in PUFAs is readily attacked by oxidation (Zaloga, 2021), and C-H reacts with ferrous ions to form a large amount of hydroxyl radicals (HO^**•**^) (Daviu et al., 2024), which define the Fenton reaction. HO^**•**^ induces peroxidation of the PUFA on the cell membrane (Balakrishnan and Kenworthy, 2024), leading to the accumulation of lipid peroxides. And enzymes such as ACSL4, LPCAT3, and LOXs convert PUFA into PL-PUFA-OOH to activate ferroptosis (Jiang et al., 2023). Authors recently ascertained that phospholipids from the tails of two PUFA groups are more likely to promote ferroptosis in A549 cells and IGROV-1 cells (Qiu et al., 2024; Figure 2).

There are four antioxidant pathways in cells; i.e., the system Xct/GSH/GPX4 pathway (Wu et al., 2024), FSP1/CoQ/NAD(P)H pathway (Wu et al., 2024), GCH1/BH4/BH2 pathway (Chuaiphichai et al., 2017), and the DHODH/CoQ pathway (Mao et al., 2021). These signal-transducing systems can reduce ROS levels and inhibit lipid peroxide production through antioxidants such as GPX4, NAD(P)H, BH4, and CoQH2. GPX4 is the fourth member of the selenium-containing GPX family, and is located in the cytoplasm and mitochondria (Miyamoto et al., 2022). GPX4 reduces oxidized glutathione (GSSG) to reduced glutathione (GSH). With the assistance of GSH, GPX4 clears membrane-lipid hydrogen peroxide products and converts them into corresponding alcohols—thus achieving anti-ferroptosis. While GPX4 is the most important anti-ferroptotic substance (Bersuker et al., 2019). CoQH2 is a free radical-scavenging antioxidant, that also inhibits ferroptosis. The mitochondrial enzyme dihydrooroate dehydrogenase (DHODH) reduces CoQ to CoQH2 (Wang and Min, 2021), and a study on heart transplantation suggested that severe ferroptosis occurred in elderly donors after transplantation, and that this was mainly due to reduced expression of DHODH, inhibition of CoQ reduction, and an exacerbation of lipid peroxidation, that collectively led to ferroptosis after heart transplantation (Zhu et al., 2024). As can be concluded from the above information, the antioxidant system within the mitochondria principally affects GPX4 and DHODH expression and location (Figure 2).

An increasing number of studies have reported that mitochondria are involved in regulating ferroptosis, oxidative stress, drug toxicity, and other stimuli in cardiomyocytes, and that all these can induce mitochondrial damage. The most obvious oxidative stress in heart disease is myocardial ischemia-reperfusion injury (MIRI), and ferroptosis constitutes the primary mode of myocardiocyte death in the middle and late stages of MIRI. ALOX15 peroxidizes AA-PE to PL-AA-OOH, and its metabolites comprise triggering factors for myocardiocyte ferroptosis. Targeted inhibition of ALOX15 or its metabolites are able to protect MIRI myocardiocytes from ferroptosis (Ma et al., 2022; Cai et al., 2023). In addition to the accumulation of mitochondria lipid metabolites that induce ferroptosis, other mitochondrial metabolic intermediates can also induce oxidative stress and ferroptosis in cardiomyocytes. Methylmalonic acid, an intermediate product of mitochondrial metabolism, enhances the interaction between NRF2 and KEAP1, and induces oxidative stress and ferroptosis that lead to myocardial injury. Hence, targeted regulation of mitochondrial metabolites effectively protects against myocardial injury (Guo et al., 2024). In addition to MIRI model, the septic cardiomyopathy model also increases oxidative stress-induced mitochondrial damage and ferroptosis. An analysis of sepsis induced cardiomyopathy showed that increasing malonylation of the mitochondrial OMM channel’s VDAC2 K46 site led to myocardial injury via mitochondrially related ferroptosis. While mutation of VDAC2 or overexpression of the deacetylase Sirtuin 5 reduce ferroptosis (She et al., 2023). In addition to the aforementioned cardiomyopathy models, there is also a classic chemotherapeutic drug, doxorubicin (DOX) that induces cardiomyopathy (DIC), and is associated with mitochondrial dysfunction and ferroptosis. In DIC, the interaction between OMM protein and GSH transporter protein reduces the content of GSH in mitochondria, weakens the antioxidant capacity of GPX4, generates mitochondrial dysfunction, and promotes ferroptosis (Ta et al., 2022). DOX is also a chelating agent of Fe^3+^, which can be reduced to DOX-Fe^2+^ in a concentration-dependent manner, thus increasing the production of HO^**•**^. This process consumes GPX4, induces lipid peroxidation of mitochondria and myocardiocyte membranes, and exacerbates ferroptosis, which creates an important cardiomyocyte death pathway in DIC (Tadokoro et al., 2023). The onset of heart disease is caused by multiple factors, and various PCDs can occur in series. Iron overload in the ferroptosis-signaling pathway results from ferritin autophagy and iron sulfur cluster autophagy. Mitochondrial quality control is related to mitochondrial autophagy. PM2.5 exposure was demonstrated to elevate the incidence of cardiovascular diseases. For example, when PM2.5 (5 mg/kg) was administered to mice via intratracheal instillation every 3 days for 4 weeks, the mice manifested myocardial hypertrophy and marked myocardial mitochondrial damage. MitoQ, a mitochondria-reducing substance, alleviate the myocardial damage and structural remodeling caused by PM2.5. Ferritin autophagy led to an imbalance in iron homeostasis at the early exposure stage. PINK1 and Parkin were then upregulated at the late stage, suggestive of that mitochondrial autophagy. Knockout of nuclear receptor coactivator 4 (NCOA4) inhibited ferritin autophagy, resulting in downregulation of DHODH and upregulation of COX2. This indicated that PM2.5 induced myocardial hypertrophy via NCOA4 mediated ferritin autophagy and mitochondrial autophagy, which led to an imbalance in iron homeostasis and a weakening of DHODH/CoQH2 antioxidant defense mechanism (Li T. et al., 2024; Figure 2).

In summary, the molecular mechanism involved in ferroptosis is closely related to antioxidant capacity, iron metabolism, and lipid metabolism in mitochondria. Targeting specific ferroptosis-signaling pathways, would protect the integrity of mitochondrial morphology and function, and constitutes a promising strategy for treating ferroptosis in various heart diseases.

### 3.2 Mitochondrial dysfunction and autophagy

Autophagy is a normal and dynamic life process in which cells use lysosomes to degrade damaged, aging, supernumerary biomolecules and organelles into basic substances to maintain a stable intracellular environment. Autophagy is considered a self-protection mechanism in the body, and both excessive autophagy and autophagic defects can lead to the onset of disease. Mitophagy is a highly conserved cellular process in eukaryotic cells that selectively clears dysfunctional or excess mitochondria through autophagic mechanisms, and thus effectively maintains the quality control of mitochondria (Hu et al., 2021). In an environment of ROS accumulation, nutrient deficiency, and cellular aging, intracellular mitochondria experience depolarizational damage and loss of mitochondrial MMP (Hu et al., 2021; Tan et al., 2022). Autophagy then recognizes and envelops damaged mitochondria, binds to lysosomes, and promotes the degradation of mitochondrial contents (Tan et al., 2022).

**FIGURE 3:**
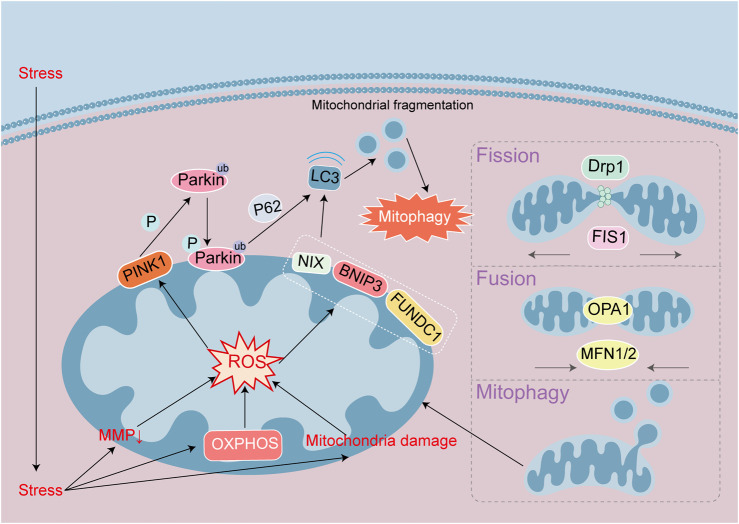
Signaling mechanisms leading to autophagy in heart disease. Autophagy maintain a stable intracellular environment. Mitophagy initiation is through the PINK1/Parkin pathway or the autophagy receptor NIX/BNIP3/FUNDC1 pathway, respectively. Parkin and NIX/BNIP3/FUNDC1 can bind to LC3 proteins to mediate autophagy. Mitochondrial damage leads to MMP decline and ROS accumulation, and mitophagy initiates. Mitochondria maintain stability through division and fusion mediated by DRP1, FIS1, OPA1 and MFN.

Mitochondrial quality control is regulated by mitochondrial autophagy and mitochondrial fission and fusion. The principal proteins involved in mitochondrial fission and fusion are DRP1, FIS1, OPA1, and MFN; and these proteins can regulate mitochondrial autophagy-related pathways (Figure 3). Although reduced autophagy is capable of inducing cellular apoptosis (Wei et al., 2021), some studies have indicated that autophagic activation engenders a rise in ferroptosis (Bi et al., 2024). Autophagy and ferroptosis jointly mediate the mechanisms underlying heart disease, and with respect to the relationship between the two. FUNDC1 (located at the OMM) is a mitochondrial autophagy receptor, GPX4 is the most important substance combatting ferroptosis. FUNDC1 interacts with GPX4 to regulate GPX4 entry into mitochondrial through the protein import system called the TOM/TIM complex. Targeting FUNDC1 can then regulate ferroptosis induced by autophagy (Bi et al., 2024). The mechanism governing heart disease onset does not comprise a single form of cell death. And myocardiocytes undergo differing degrees of damage at different stages. Therefore, disparate PCDs can be analyzed from studied through mitochondria in the same disease. DRP1 is located in the cytoplasm and can be recruited to the OMM by FIS1 and MFF, and investigators have shown that in aging cardiomyocytes, the downregulation of DRP1 led to insufficient mitochondrial autophagy and promoted apoptosis (Wei et al., 2021). In multiple heart failure models, key proteins or enzymes have been adopted to target different signaling pathways that promote DRP1-dependent mitochondrial autophagy in order to prevent and alleviate heart failure (Forte et al., 2023; Xu et al., 2023; Yan M. et al., 2024). In addition to changes in the expression levels of DRP1, the activity and subcellular localization of DRP1 can also affect mitochondrial autophagy. For example, high-fat stimulation promoted the phosphorylation of DRP1 Ser616 in the human heart, leading to mitochondrial translocation, augmented mitochondrial division, and increased mitochondrial autophagy (Tong et al., 2023). In addition, OPA1 and MFN are capable of promoting mitochondrial fusion. In the mouse model of hypertrophic cardiomyopathy induced by FARS2 gene defects, the levels of MFN1, MFN2, and OPA1 were downregulated, but DRP1 recruitment increased, leading to increased mitochondrial fragmentation and initiating mitochondrial autophagy, which is one cause of myocardial injury (Li B. et al., 2024). Hence, maintaining mitochondrial quality control is closely related to mitochondrial autophagy. In different heart disease models, mitochondrial autophagy is dynamically regulated through changes in mitochondrial fission and fusion that maintain and protect heart function.

The mitochondrial autophagy pathway can be divided into ubiquitin-dependent and ubiquitin-independent pathways. The key proteins involved in the ubiquitin-dependent pathway are PINK1 and Parkin (Figure 3). PINK1 is a serine/threonine kinase located on depolarized mitochondria, while Parkin is an E3 ubiquitin ligase that catalyzes the transfer of ubiquitin to mitochondrial substrates. Under physiological conditions, the precursor protein of cytoplasmic PINK1 targets and enters mitochondria and then is cleaved by proteases from the mitochondrial matrix and IMM before release into the cytoplasm and hydrolysis by ubiquitin proteasomes (Jiménez-Loygorri et al., 2024). However, in pathological conditions, when the MMP falls, the entry of PINK1 precursor protein into the mitochondria is blocked, and the accumulated PINK1 precursor forms a dimer on the OMM that undergoes phosphorylation and activation. The activated PINK1 subsequently recruits Parkin from the cytoplasm to the OMM and activates its E3 enzyme activity. Parkin then activates ubiquitination of the mitochondrial outer membrane protein, and the ubiquitin chain is phosphorylated by PINK1. The phosphorylated ubiquitin modified the outer membrane proteins for recognition by autophagic proteins, and autophagy is then activated. During the physiological process of mouse aging, mitochondrial autophagic proteins in various tissues and organs increase. Mitochondrial autophagy enhances the mediation of the PINK1/Parkin pathway by changes in MMP, with PINK1-induced phosphorylation of ubiquitin at the Ser65 site (Jiménez-Loygorri et al., 2024).

Non-ubiquitin-dependent mitochondrial autophagy is mediated by the mitochondrial autophagic receptors NIX, BNIP3, FUNDC1, and others located on the OMM that contain a conserved LC3 binding domain (LIR) (Figure 3). These receptors directly bind to the autophagy-related protein LC3 through the LIR motif, initiating autophagy. Non-ubiquitin-dependent receptors remain inactive on the outer membrane of the mitochondria until mitochondrial autophagy is triggered, regulating phosphorylation/dephosphorylation processes through dimerization. For example, programmed mitochondrial autophagy is associated with the maturation of myocardiocytes (Baechler et al., 2019). The mitochondrial autophagy receptor BNIP3/NIX located on the OMM, is a key factor that affects mitochondrial function after hypoxia, ischemia, or reperfusion. This receptor combines with mitochondrial binding sites to regulate mitochondrial autophagy, causing damage to the mitochondria (Gok et al., 2023). The mechanism underlying FUNDC1 receptor action has become an area of intense interest in recent years with respect to mitochondrial autophagy in heart disease. In an obesity-related study, FUNDC1 interacted with the related ubiquitin ligase complex receptor subunit to regulate IP3R2 receptor degradation, prevent calcium overload, and alleviate myocardial lipotoxic damage by mitochondrial autophagy (Ren et al., 2020). Under hyperlipidemia conditions, FUNDC1 deficiency also regulated ACSL4-mediated ferroptosis, leading to cardiac remodeling and dysfunction (Pei et al., 2021). FUNDC1 is an autophagy receptor that engenders a cascade of cellular autophagic and ferroptosis events. After ischemia-reperfusion injury, neutrophil extracellular traps activate ferroptosis by blocking the activation of FUNDC1-dependent mitochondrial autophagy (Chu C. et al., 2023). In fibrosis damage, FUNDC1 promotes the entry of GPX4 into mitochondria, thus regulating ferroptosis (Bi et al., 2024). Numerous studies have revealed that ferroptosis and mitochondrial autophagy interact with mitochondrial damage.

In summary, mitochondrial autophagy is a downstream event that is influenced by mitochondrial growth, division, and fusion. By regulating the targets of upstream signal transduction pathways of mitochondrial division and fusion, or mitochondrial autophagic signal transduction pathways, mitochondrial autophagy protects the integrity of mitochondrial morphology and function and treats various heart diseases.

### 3.3 Mitochondrial dysfunction and apoptosis

Apoptosis is an active process of cell death that is regulated by a series of genes, does not release cellular contents to the surrounding environment, and is different from cellular necrosis (Singh et al., 2019). Apoptosis involves a series comprising gene activation, expression, and regulation, and is characterized by membrane asymmetry, loss of attachment, nuclear membrane fragmentation, DNA fragmentation and decay, and the formation of apoptotic bodies with specific structures. The activation of cellular apoptosis can be divided into endogenous and exogenous signaling pathways, the exogenous death receptor pathway begins with the binding of specific death receptors to ligands, and the endogenous apoptosis is a pathway that interacts with mitochondria (Newton et al., 2024). Apoptosis defects lead to the occurrence of cell death disorders (Deen et al., 2024). Overactivation of apoptosis leads to activation of relevant pro-apoptotic genes and proteins, which increase cell death (Zhu et al., 2019). Therefore, studying the apoptotic signaling pathway can provide effective therapeutic targets for various diseases.

**FIGURE 4:**
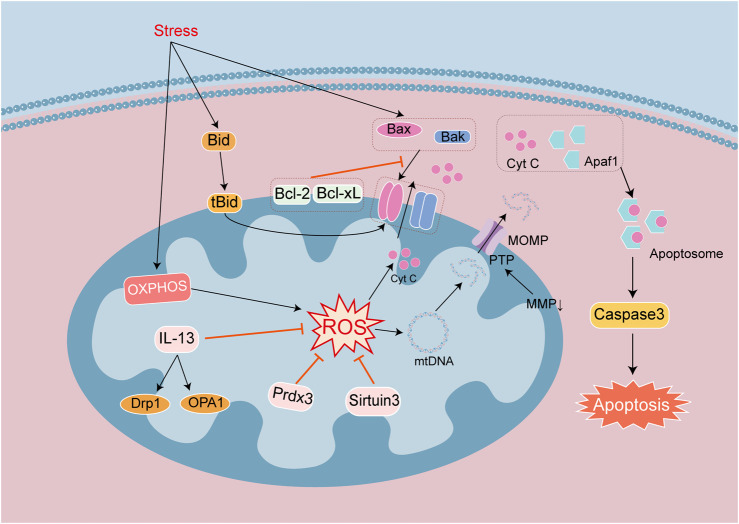
Signaling mechanisms that lead to apoptosis in heart disease. Apoptosis is a process of cell death regulated by a series of genes. Endogenous apoptotic signaling is executed and regulated by the Bcl-2 family of proteins. They are divided into anti-apoptotic proteins Bcl-2 and Bcl-xL, and pro-apoptotic proteins Bak, Bid and Bax. Antiapoptotic proteins can inhibit the release of Cyt c. Apoptotic signals promote the dimer formation of proapoptotic proteins, which lead to form transmembrane pores in the mitochondrial surface. The integrity of the mitochondrial outer membrane is broken by decreasing MMP, changes in MOMP and open PTP channels. Cyt c released into the cytoplasm binds to Apaf1 to form Apoptosome, which activates Caspase 3 to mediate apoptosis. Mitochondrial damage leads to increase ROS and mtDNA damage. The release of Cyt c and mtDNA into the cytosol exacerbates cell apoptosis.

The endogenous apoptotic signaling pathway is executed and regulated by the proteins of the Bcl-2 family, which includes antiapoptotic proteins Bcl-2 and Bcl-xL, and the proapoptotic proteins Bak, Bid, and Bax (Dadsena et al., 2024). The antiapoptotic proteins Bcl-2 and Bcl-xL are located in the OMM and can inhibit the release of cytochrome c (Cyt c), while the proapoptotic proteins Bak, Bid, and Bax are free in the cytoplasm. Under physiologic circumstances, Bcl-2 and Bcl-xL form heterodimers with Bax and Bak to maintain the integrity of the mitochondrial outer membrane (Xu et al., 2020), and after receiving apoptotic signals, they translocate into mitochondria and form transmembrane pores on the mitochondrial surfaces. Mitochondrial dysfunction leads to a diminution in MMP and rise in membrane permeability. Mitochondrial Cyt c is released to the cytoplasm and binds with apoptotic enzyme activators to form ATP-dependent apoptotic complexes. Procaspase-9 is recruited to the apoptotic complex and is activated after self-cleavage, mobilizing caspase-3 and initiating the caspase-cascade reaction. Caspase-3 cleaves over 100 substrates in cells, including α-tubulin, actin, PARP, and lamin, ultimately leading to cell apoptosis (Xu et al., 2024). Protein tyrosine phosphatase (PTP) is embedded between the OMM and IMM, forming a mitochondrial permeability transition pore (MPTP) that allows small molecules to pass through. When the mitochondrial matrix experiences high osmotic pressure, the MPTP opens allowing the mitochondrial permeability transition; and this disrupts the IMM and leads to sustained mitochondrial depolarization. Bax and Bak are activated by infiltrating the OMM, releasing apoptotic factors into the cytoplasm, triggering mitochondrial outer membrane permeability (MOMP), and forming apoptotic bodies, which promote apoptosis (Dadsena et al., 2024; Figure 4). Research has shown that MOMP appeared during cellular aging and activated the cGAS-STING pathway by releasing mtDNA and driving the production of age-related secretory phenotypes. This suggests that cellular aging leads to increased apoptosis, mitochondrial damage, and the release of mtDNA to promote aging. Changes in MMP also lead to the release of contents, such as mtDNA, but Cyt c-mediated cellular apoptosis occurs earlier than the activation of the cGAS-STING pathway by mtDNA (Jiménez-Loygorri et al., 2024), with the timing of activation related to the degree of mitochondrial damage.

In the study of heart disease models, apoptosis often not only occurred in acute injury, such as MIRI, sepsis cardiomyopathy, viral infection cardiomyopathy, acute myocardial infarction (AMI), and DIC, but also in chronic diseases complicated by cardiomyopathy, including diabetes cardiomyopathy (DCM) and heart failure. And mitochondrial dysfunction is the research focus of these disease models. Numerous investigators have examined the production of ROS in mitochondria, as causing mitochondrial fission and fusion disorders, and inducing mitochondrial autophagy. Mitochondria dysfunction promotes apoptosis, participates in regulating the signaling pathways of mitochondrial antioxidants and mitochondrial growth, and has become a therapeutic target for studying cellular apoptosis. As noted earlier, the deacetylase family repairs mtDNA and reduces damage, and Sirtuin 3 is one member of the family that facilitates mitochondrial energy homeostasis, antioxidant activity, proliferation, and DNA repair. Mitochondrial Sirtuin 3 has been shown to be activated in different cardiac disease models (Yang Z. et al., 2024), and Sirtuin 3 can mediate the deacetylation of PRDX3, alleviate mitochondrial oxidative damage and lipid and ROS accumulation to inhibit myocardiocyte apoptosis (Wang Z. et al., 2020). It is also able to reduce mitochondrial iron accumulation, and maintain the synthesis of iron sulfur cluster proteins and mitochondrial iron balance to alleviate myocardiocyte apoptosis (Gao et al., 2023). In addition to antioxidant research, the relationships between mitochondrial integrity, mitochondrial fission and fusion, autophagy, and cell apoptosis have also been validated in several reports. In MIRI, mitochondrial integrity can be maintained and myocardiocyte apoptosis can be reduced by regulating the targets of the Keap1/NRF2 pathway (Zhang L. et al., 2020). IL-13 is the principal regulatory factor for mitochondrial biogenesis. In the model of septic cardiomyopathy, IL-13 treatment can restore mitochondrial function and morphology, and reduce myocardiocyte apoptosis (Guo et al., 2021). The above two disease models act on different targets, but both of them maintain mitochondrial functional integrity so as to reduce myocardiocyte apoptosis.

The integrity of mitochondrial function is closely related to mitochondrial autophagy, and the degree of mitochondrial autophagy is used to determine the progression of cell apoptosis. As stated previously, reduced autophagy can induce cell apoptosis. For example, the downregulation of DRP1 in aged mice leads to insufficient mitochondrial autophagy and promotes apoptosis in aging cardiomyocytes (Wei et al., 2021). Drug activation of OPA1 mediated mitochondrial autophagy reduce myocardiocyte apoptosis in AMI model (Xin and Lu, 2020). A study about DIC model draw the same conclusion that autophagy and apoptosis of cardiomyocytes were negatively correlated. Myocardiocyte apoptosis is regulated through the AMPK/mTOR axis (Zhang et al., 2023). However, not all mitochondrial autophagy is negatively correlated with apoptosis, which may be due to disrupted compensatory mechanisms. In a viral cardiomyopathy model, viral infection activated calpain lysis and calcineurin A, which led to dephosphorylation of the Ser637 site of Drp1. This precipitated excessive mitochondrial fission, causing mitochondrial dysfunction and inducing cardiomyocyte apoptosis (Shi et al., 2022). In addition, one study has reported that in DOX-induced heart failure models, the transplantation of mesenchymal stem cell mitochondria inhibited excessive mitochondrial autophagy and alleviated myocardiocyte apoptosis (Jin et al., 2024). There are various conclusions from the papers regarding the relationship between mitochondrial autophagy and cellular apoptosis. As for whether mitochondrial autophagy is beneficial or harmful, this dichotomy is closely related to the degree of activation of mitochondrial autophagy. Under physiologic conditions, there is a balance between autophagy and apoptosis. When autophagy is low, the degree of autophagy is inversely proportional to apoptosis, and it is then necessary to activate autophagy to inhibit apoptosis. However, when autophagy is excessive, the degree of autophagy is proportional to apoptosis, and it is necessary to inhibit both autophagy and apoptosis.

In summary, the mitochondrial pathway of cellular apoptosis occurs via the Bcl-2 family in forming mitochondrial membrane pores and releasing pro-apoptotic factors such as Cyt c, which initiates cell apoptosis. However, cell apoptosis can be maintained by inhibiting mitochondrial morphology and function, and this is related to mitochondrial autophagy. Regulating mitochondrial autophagy can therefore affect cellular apoptosis and may be useful in treating myocardial apoptosis in various heart diseases.

### 3.4 Mitochondrial dysfunction and pyroptosis

Cellular pyroptosis is an inflammatory PCD of the cell membrane pores formed by the gasdermin protein family. The degree of pyroptotic injury is between apoptosis and necrosis with nuclear condensation and cleavage. Cellular pyroptosis is activated by caspase-1. The formation of pores in the gasdermin protein on the cell membrane can lead to loss of cellular integrity, increased permeability, and release of cellular contents. The release of inflammatory factors leads to an inflammatory response. Ultimately, the cell membrane undergoes rupture and dissolution to induce cell pyroptosis.

**FIGURE 5:**
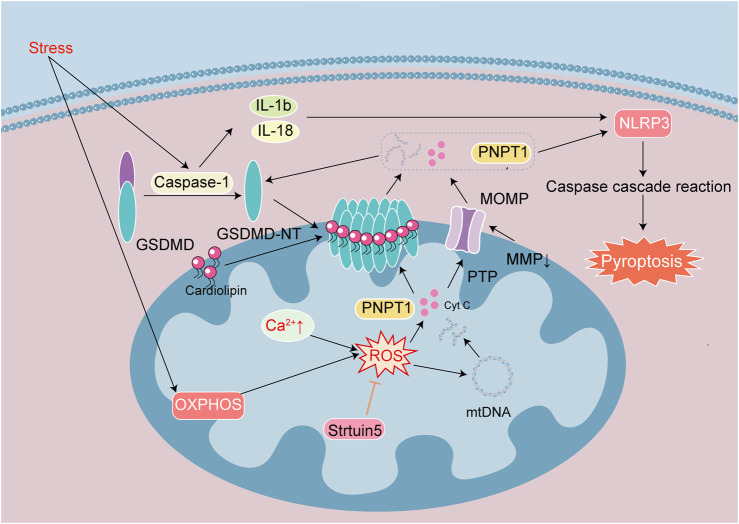
Signaling mechanisms leading to pyroptosis in heart disease. Pyroptosis is one of cell death induced by inflammatory effect. Stress activates Caspase-1, GSDMD, IL-1β and IL-18. The active fragment GSDMD-NT binds cardiolipin on the membrane to form a special pore, and MOMP causes MMP decrease and PTP channel opening. They cause the loss of cellular integrity and the release of cellular contents such as ROS, Cyt c, mtDNA, PNPT 1. OXPHOS and Ca^2+^ overload caused increased ROS to exacerbate mtDNA damage. Release of the exonuclease PNPT1 degrades the cytoplasmic mRNA, amplifying the downstream inflammatory response and aggravating pyroptosis. Sirtuin 5 reduce ROS and stabilize the mitochondrial environment.

Gasdermin D (GSDMD) is the executor of cell pyroptosis. GSDMD is composed of N-terminal (GSDMD-NT) and C-terminal domains (GSDMD-CT). GSDMD-NT manifests inherent drilling activity, and in its full-length state GSDMD-CT can inhibit the activity of GSDMD-NT. When pathogens enter the body, they release specific chemicals that are recognized by the corresponding receptors on immune cells, triggering an immune response. Pathogens then bind with apoptosis-related proteins to recruit procaspase-1 and drive the formation of inflammasomes. Inflammasomes activate caspase-1 to become mature lysed caspase-1, IL-1β and IL-18 are activated into mature proteins, promoting GSDMD to produce GSDMD-NT. GSDMD-NT is able to bind membrane cardiolipin, form pores in the plasma membrane, and initiate MOMP. Pores and MOMP subsequently lead to the release of cytokines such as IL-1β and IL-18 and various cytoplasmic contents, ultimately resulting in membrane rupture and cellular pyroptosis (Toldo and Abbate, 2024; Figure 5).

The effects of mitochondrial damage include decreased mitochondrial count, loss of MMP, weakened OXPHOS, accumulation of ROS, release of mitochondrial proteins and mtDNA in the matrix, mitochondrial autophagy. The above effects increase formation of inflammasomes and lead to activation of GSDMD. GSDMD-NT is localized in the mitochondria during the early stages of pyroptosis. When mitochondria are damaged, the mitochondrial membrane undergoes depolarization and the activated GSDMD-NT translocates to the mitochondria, leading to form pores on their membranes, increase membrane permeability, release of Cyt c and mtDNA into the cytoplasm. Mitochondrial damage promotes GSDMD activation, which in turn exacerbates mitochondrial damage and creates a vicious cycle (Ye et al., 2022). Mitochondrial damage in cell pyroptosis is independent of the Bcl-2 family and depends upon the binding of GSDMD-NT to cardiolipin. Mitochondrial damage, inflammatory cytokine release, and inflammasome activation can all be inhibited by knocking out the cardiophospholipid synthase gene or phospholipid flipping enzyme-3 gene that produces and transfers cardiophospholipids to the OMM (Miao et al., 2023). Therefore, mitochondrial damage plays a critical role in cell pyroptosis. The mitochondrial dysfunction caused by GSDMD-NT occurs before cell death, while inhibition of mitochondrial damage alleviates the pyroptotic process.

GSDMD-mediated mitochondrial damage leads to the release of the 3′-5′ end exonuclease PNPT1 into the cytoplasm, degrading mRNA globally, and this exacerbates cell pyroptosis and amplifies downstream inflammatory responses. ROS have long been considered key signaling molecules for cell apoptosis. ROS trigger the activation of NLRP3 inflammasomes and oxidize GSDMD, which further enhance the activation of caspase-1, induce the efficiency of GSDMD lysis, and subsequently trigger cell pyroptosis via IL-1β and IL-18 maturation (Liu et al., 2022). The activation of caspase-1 inhibits physiological mitochondrial autophagy, leading to further increase in mitochondrial damage and ROS production. The activation of NLRP3 requires Ca^2+^ participation, Ca^2+^ overload damages mitochondria and increases ROS production. There is thus a widespread crosstalk between cellular pyroptosis and apoptosis. Inflammatory stimulation also activate caspase-3, and mitochondrial damage by inflammasomes induce MOMP. PTP and GSDMD pores occur simultaneously, increasing mtDNA release and ROS production (Figure 5).

Cellular pyroptosis is closely associated with inflammation, and myocardial damage that triggers inflammation is closely associated with cellular pyroptosis. Most researchers using heart disease models focus on the regulation of the inflammasome NLRP3, which is linked to the downstream cascade of mitochondrial damage. The activation of NLRP3 and caspase-1 is reduced through various signal transduction pathways and cell pyroptosis is inhibited (Shi et al., 2021; Liu et al., 2022; Liu et al., 2024; Wang et al., 2024; Zhang et al., 2024). Although there is a obvious inflammatory response in the early stage of MIRI, MIRI is alleviated after specific knockout of GSDMD in myocardiocytes, indicating that cell pyroptosis constitutes an important link in early MIRI-induced myocardial injury (Shi et al., 2021). Negative regulation of NLRP3 by ubiquitination prevents myocardiocyte pyroptosis and myocardial injury (Liu et al., 2024). Although there are limited studies on mitochondrial damage in the early stages of pyroptosis, some researchers still suggest that intervention during the mitochondrial damage stage would inhibit pyroptosis. In DIC models, DOX not only induced inflammation to activate caspase-1 and GSDMD, but also mediated mitochondrial damage and perforation in cardiomyocytes via the autophagy receptor BNIP3 (Ye et al., 2022). DOX-induced myocardial injury inhibited levels of mitochondrial autophagy related proteins and increased pyroptosis related proteins. Promoting the upregulation of AMPK-ULK1-FUNDC1 pathway proteins, facilitates mitochondrial autophagy, reduces ROS levels, improves MMP, alleviates mitochondrial damage, and reduces DOX-induced pyroptosis (Zhao et al., 2024). In addition to their relation with mitochondrial autophagy, modifications of mitochondrial proteins are also involved in regulating cellular pyroptosis. In the DCM model, the mitochondrial proteins desuccinylase and succinylase Sirtuin 5 are activated by transcription factors. Sirtuin 5 is a member of the deacetylases family. Sirtuin 5 maintains the stability of glutathione S-transferase P-lysine malonylation and its modifications and improves mitochondrial function, thereby alleviating myocardiocyte pyroptosis (Wei et al., 2024). In the process of cell pyroptosis, the damage and release of inflammatory-mediator mtDNA can also promote pyroptosis. In a chronic myocardial ischemia model, activation of caspase-1 through mtDNA damage promoted cellular pyroptosis (Wang X. et al., 2020).

In summary, it can be concluded that mitochondrial damage occurs in the early stages of cell pyroptosis and progressively intensifies to participate in the subsequent vicious cycle. A majority of current research analyses focus on the regulation of GSDMD-NT and NLRP3 activation, and mitochondrial damage occurs upstream of this activation. Maintaining the integrity of mitochondrial morphology, structure, and function in the early stages of pyroptosis appears to inhibit the onset of cellular pyroptosis.

### 3.5 Mitochondrial dysfunction and cuproptosis

**FIGURE 6:**
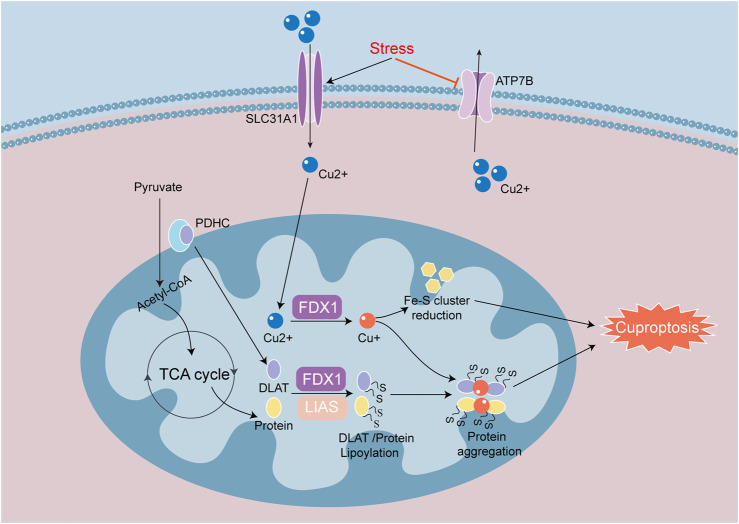
Signaling mechanisms of cuproptosis in heart disease. Cuproptosis is a process of cell death mainly caused by the reduction of Fe-S cluster proteins and the aggregation of lipoylated proteins. SLC31A1 and ATP7B regulate intracellular copper in and out. Iron redox protein 1 (FDX 1) and fatty acid synthase (LIAS) are involved in the regulation of thiylation of DLAT and TCA cycle proteins. FDX1 converts Cu^2+^ to Cu^+^, suppresses the synthesis of Fe-S cluster proteins and induces cuproptosis. When Cu^+^ overaccumulates in mitochondria, Cu^+^ will bind to lipoylated proteins and induce heteromerization of lipoylated proteins, causing proteotoxic response to cuproptosis.

Cuproptosis is a novel cell death mode, which is closely related to an increase in copper ion concentration and targets TCA cycle proteins lipoylated (Tang et al., 2022). The input and output of copper are regulated by the membrane proteins SLC31A1 and ATP7B (Tsvetkov et al., 2022). Iron redox protein 1 (FDX1), DLAT, and lipoate synthase (LIAS) are key genes involved in cuproptosis. FDX1 is an upstream regulatory factor for cuproptosis, which is involved in regulating the thioacylation of DLAT. FDX1 converts Cu^2+^ to the more toxic Cu^+^, leading to inhibition of Fe-S cluster protein synthesis and the induction of cell death (Tsvetkov et al., 2022). FDX1 is a highly promiscuous mitochondrial reductase, and knockout of FDX1 or enzymes related to acylation prevents cuproptosis. In addition, knockout of FDX1 reduces NADH production, Fe-S cluster biogenesis and mitochondrial respiration (Zulkifli et al., 2023). DLAT is one of the components of the pyruvate dehydrogenase complex (PDHC) that catalyzes the decarboxylation of pyruvate in the TCA cycle in order to produce acetyl CoA. Elesclomol, which is a copper ion carrier, transports Cu^2+^ into cells, and is used to aggravate cuproptosis. When Cu^2+^ accumulates excessively in cells that rely on mitochondrial respiration, Cu^2+^ binds to lipoylated DLAT, which induces DLAT heteropolymerization. This action subsequently leads to the downregulation of Fe-S cluster protein expression and an increase in insoluble DLAT (Figure 6). The above process then ultimately leads to cytotoxicity and cell death (Tsvetkov et al., 2022).

In one study identified Cu^2+^ as a key factor in the development of cardiovascular diseases, including MIRI, heart failure, atherosclerosis and arrhythmia. Ube2d3 was identified as a key cuproptosis related gene associated with the development of myocardial infarction, which promoted p62 ubiquitination and exacerbated the damage to autophagic flow (Yang M. et al., 2024). In addition to myocardial infarction models, cuproptosis related studies have also been conducted in septic cardiomyopathy models. The expression of cuproptosis related genes was significantly changed, indicating that cuproptosis was significant in sepsis-induced myocardial injury. Cyt c oxidase 11 copper chaperones (COX11) is one of the more important genes in cuproptosis. The reduction of COX11 unable to sustain copper homeostasis in myocardial mitochondria suggests that cuproptosis is closely correlated with changes in mitochondrial function (Yan J. et al., 2024). In addition, there are reports of cuproptosis and mitochondrial dysfunction in DCM. In DCM models, sustained hyperglycemia promoted advanced glycation end products (AGEs) and copper in mouse blood, accompanied by cardiac dysfunction. The increase in AGEs further led to diminished protein thioacylation, accompanied by drop in mitochondrial oxidative respiration. Moreover, AGEs promoted the upregulation of related transcription factors, increased the entry of copper ions into cells, disrupted mitochondrial functioning, and promoted cuproptosis (Huo et al., 2023). There is currently a limit of studies on cuproptosis in heart disease, and investigations into cuproptosis as a potential interventional target remain in nascent stages and require further exploration.

In summary, cuproptosis is one of the cell death modes that induces mitochondrial dysfunction. Research on the pathogenesis of cuproptosis remains unclear. As an unfamiliar cell death mode, targeting myocardiocyte mitochondria in order to inhibit cuproptosis is expected to be a strategy for treating various heart diseases in the future.

## 4 Conclusion and perspectives

There have been literature reports on the treatment of heart failure by transplanting human umbilical cord mesenchymal stem cell mitochondria (MSC Mito). In a mouse heart failure model, MSC Mito transplantation was performed and human specific mtDNA was detected to confirm successful transplantation. These investigators ascertained that MSC Mito transplantation exerted cardioprotective effects by restoring ATP production and reducing excessive AMPK/mTOR-mediated autophagy. In addition, in rat models of AMI, MSC Mito transplantation was performed by short-term intravenous administration or long-term oral administration. This modality reduced the infarct area by 35%, restored heart function by 20%, and reduced apoptotic cells by over 50% (Jin et al., 2024). Isolated mitochondria used in the treatment of MIRI was proved intact function. Mitochondria can internalize into different receptor cells, and can be injected externally to alleviate myocardiocyte damage (Jia et al., 2022). Different PCDs lead to mitochondrial damage ultimately, thus to control mitochondrial quality achieves the purpose of treating cardiomyocyte injury. However, the toxic side effects in mitochondrial transplantation are poorly understood and are still solely evaluated at the animal model level. The application of mitochondrial transplantation in clinical practice therefore necessitates further investigation.

**FIGURE 7:**
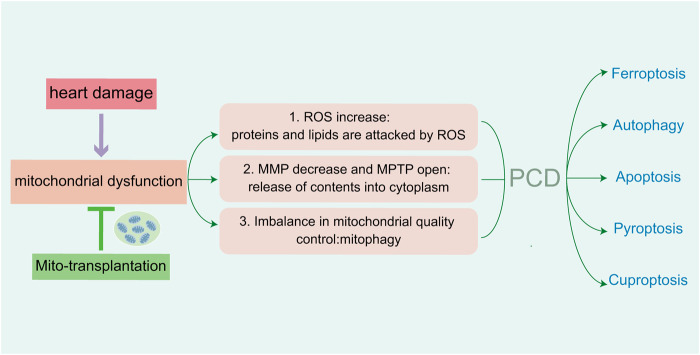
Injury of cardiomyocytes cause mitochondrial dysfunction, which mediate five PCDs in heart damage. The increasing of ROS attack related proteins and lipids. Abnormal MMP and channel-open release the contents of mitochondria. Imbalance of mitochondrial quality control lead to mitochondrial autophagy. Reducing the above aspects can alleviate mitochondrial dysfunction, as well as PCD-mediated myocardial injury.

PCD and mitochondrial function are highly correlated. In various basic research studies on heart diseases, mitochondria regulated various targets of PCD in cardiomyocytes, and this alleviated mitochondrial dysfunction by maintaining mitochondrial morphology and improving mitochondrial function. The first aim in these analyses is to maintain mitochondrial antioxidant balance. The second is maintaining the integrity of mitochondrial membranes. A final requisite is required to control mitochondrial quality. Different routes of PCD in myocardiocytes can cause mitochondrial damage via different targets. In addition, mitochondria participate in different PCDs by regulating specific targets, exogenous mitochondria treatment improves myocardiocyte health and protects cardiac function (Figure 7). This avenue has stimulated novel approaches and directions for the clinical treatment of cardiovascular diseases, but it generates side effects such as prognosis and rejection reactions. As underlying mechanisms regarding the role of mitochondria in heart disease remain unclear, further examinations are sorely needed. The application of exogenous mitochondria treatment is currently only in the animal experimental stage, and more investigations are required for future clinical treatment.

## Glossary

PCD: Programmed Cell Death
OMM: Outer Mitochondrial Membrane
IMS: Intermembrane Space
IMM: Inner Mitochondrial Membrane
ETC: Electron Transfer Chain
OXPHOS: Oxidative Phosphorylation
TCA cycle: Tricarboxylic Acid cycle
mtDNA: mitochondrial DNA
nDNA: nuclear DNA
tRNA: transfer RNA
rRNA: ribosomal RNA
ROS: Reactive Oxygen Species
ULK1: Unc-51 like autophagy activating kinase 1
BNIP3: Adenovirus E1B 19 kDa interacting protein 3-like
NADH: Nicotinamide Adenine Dinucleotide - Hydrogen
FADH2: Flavin Adenine Dinucleotide Hydrogen Donor
ANT1: Adenine Nucleotide Translocator 1
DRP1: Dynamin-Related Protein 1
FIS1: Fission 1
MFF: Mitochondrial Fission Factor
MFN1/2: Mitofusin1/2
OPA1: Optic atrophy type 1
FARS2: Phenylalanyl-tRNA synthetase
MCU: Mitochondrial Calcium Uniporter
FUNDC1: FUN14 domain-containing protein 1
IP3R2: Inositol 1,4,5-Trisphosphate Receptor Type 2
PRDX3: Peroxiredoxin 3
SO2/3-PRDX3: Peroxide Peroxiredoxin 3
PUFAs: Polyunsaturated Fatty Acid
**HO** ^**•**^: Hydroxyl Radicals
ACSL4: Acyl-CoA Synthetase Long-chain family member 4
LPCAT3: Lysophosphatidylcholine Acyltransferase 3
LOXs: Lipoxigenase
GPX4: Glutathione Peroxidase 4
GSH/GSSG: L-Glutathione/Glutathione Oxidized
FSP1: Ferroptosis-Suppressor Protein 1
CoQ/CoQH2: Coenzyme Q/Reduced Coenzyme Q10
GCH1: GTP cyclohydrolase 1
BH4/BH2: Tetrahydrobiopterin/Dihydrobiopterin
DHODH: Dihydroorotate Dehydrogenase
MIRI: Myocardial Ischemia-Reperfusion Injury
ALOX15: Arachidonate-15-Lipoxygenase
NRF2: Nuclear Factor Erythroid 2-Related Factor 2
KEAP1: Kelch-like ECH-Associated Protein 1
VDAC2: Voltage-Dependent Anion Channel 2
DOX: Doxorubicin
DIC: Doxorubicin Induced Cardiomyopathy
PINK1: PTEN-Induced Putative Kinase 1
NCOA4: Knockout of Nuclear Receptor Coactivator 4
TOM/TIM: Translocase of the Outer Membrane/Translocase of the Inner Membrane
LC3: Light Chain 3
TMEM11: Transmembrane Protein 11, Mitochondrial
Cyt C: Cytochrome C
PTP: Protein Tyrosine Phosphatase
MPTP: Mitochondrial Permeability Transition Pore
MOMP: Mitochondria Outer Membrane Permeability
AMI: Acute Myocardial Infarction
DCM: Diabetes Cardiomyopathy
GSDMD: Gasdermin D
Plscr3: Phospholipid Scramblase 3
PNPT1: Polyribonucleotide Nucleotidyltransferase 1
NLRP3: NOD like Receptor thermal Protein domain associated Protein 3
FDX1: Feredoxin-1
DLAT: Dihydrolipoamide S-acetyltransferase
LIAS: Lipoylsynthase, Mitochondrial
COX11: Cytochrome c Oxidase Subunit 11
MSC Mito: Mesenchymal Stem Cell Mitochondria
